# Peer-Assisted Learning Improves Confidence and Educational Outcomes: Evidence from a Randomised Control Trial

**DOI:** 10.1007/s40670-025-02554-x

**Published:** 2025-11-08

**Authors:** Ferdinand J. O. Boucher, Bella A. Magner-Parsons, David MacDonald, Tim W. Fawcett, Ellis J. G. Langley, Amanda Pocklington

**Affiliations:** 1 University of Exeter Medical School, Exeter, EX2 4TH England; 2 Centre for Biological Diversity, School of Biology, University of St Andrews, Sir Harold Mitchell Building, St Andrews, KY16 9TH Scotland; 3 Department of Psychology, University of Exeter, Exeter, EX4 4QG England

**Keywords:** Peer-assisted learning (PAL), Near-peer-assisted learning (NPAL), Higher education, Weekly test scores, Randomised control trial (RCT), Student confidence

## Abstract

The positive impact Peer-Assisted Learning (PAL) and Near-Peer-Assisted Learning (NPAL) programmes have on student engagement, progression, and retention is supported by a wealth of previous studies and is reflected in the widespread adoption of (N)PAL programmes across higher education institutions. However, controlled investigations of the efficacy of (N)PAL programmes using quantifiable outcomes are limited. In this study, a within-participant randomised control trial was used to assess weekly learning and student confidence. Across four weeks, all participants attended (in counterbalanced order) an introductory (pre-lecture) NPAL session, a review (post-lecture) NPAL session, both introductory and review sessions, or a no-session control. Our data indicate that student attendance at the introductory NPAL session had a positive effect on their weekly test score (*p* = .027)*,* response speed (*p* = .050) and self-reported confidence score (*p* = .006) in the same week that the session was delivered, whereas there was no detectable effect of attending the review NPAL session. These findings provide experimental evidence for a measurable, beneficial effect of NPAL programmes on student confidence and educational outcomes, suggesting a simple intervention can have positive impacts within a short space of time. Our trial provides a framework for higher education institutions to use when evaluating their own (N)PAL programmes and may help to inform the design and implementation of such programmes throughout the global higher education sector.

## Introduction

Peer-assisted learning (PAL) and near-peer-assisted learning (NPAL) has been widely reported to enhance student learning experiences [1–4]. (N)PAL diverges from traditional academic-led teaching approaches, by utilising peers (other students in the same class) or ‘near peers’ (other students at a slightly more advanced stage of study) as mentors (sometimes referred to as ‘tutors’ or ‘facilitators’) to supplement traditional learning [5]. This differs from traditional teacher-led learning environment as it promotes a higher degree of collaboration and emphasis on mutual learning to achieve a common goal. Whilst these (N)PAL sessions retain an element of traditional didactic teaching, the near-peer teaching elements offers a more relaxed and nurturing environment for the students to engage in and help booster their learning in the field being taught. Neer-peer and peer tutors are recruited for their academic strength and confidence in teaching the learning material, but also for their understanding around establishing a strong learning environment, embedding appropriate learning theories at the heart of their teaching sessions [6]. Students’ participation in academic (N)PAL programmes, where the focus is on promoting development of academic skills, understanding of curricula, and preparing for assessment, has been linked to a broad range of positive indices, including higher average grades, lower failure rates, and higher course retention and graduation rates [7, 8]. Students are motivated to engage in these programmes in search of academic success as well greater, more holistic learning outcomes centred around advancing their learning behaviour and work patterns. These learning aspects can then be translated further in their academic careers, not least for their future years in higher education. This has resulted in peer programmes being adopted by higher education institutions across the world as a cost-effective addition to core teaching practices that may benefit both student participants and student peer mentors [9]. To evaluate the efficacy of these programmes more fully, it is important to conduct educational research that measures the academic attainment of students who participate in them.

Despite widespread adoption of (N)PAL programmes in higher education, many evaluative investigations rely on qualitative data obtained through observational studies, evaluation surveys and focus groups, with quantitative measures assessing performance in time-disparate mid-year and/or end-of-year assessments [10–12]. These studies have identified associations between (N)PAL participation and increased student confidence, academic engagement, academic achievement, and social confidence. While qualitative data provide a rich experiential account of (N)PAL programme efficacy, the lack of quantitative findings precludes the ability to investigate effect sizes systematically and thus assess the utility of (N)PAL programmes in educational contexts [13]. Furthermore, most prior studies that reported quantitative research findings did not involve randomised allocation to different study groups and so cannot exclude the possibility that higher-achieving or more motivated students are more likely to attend (N)PAL programmes, rather than there being a direct beneficial effect of the programme [7, 8]. Finally, significant methodological and measurement heterogeneity, alongside poor study quality and limited experimental research, have made cross-study comparison difficult [4, 7, 8].

A small selection of studies stands out as offering a more robust evidence base to explore how participation in (N)PAL programmes causally affect academic performance. A randomised control trial (RCT) assessing effects of an undergraduate mentoring programme within a nursing degree course in the USA demonstrated that, compared to controls, students receiving PAL sessions self-reported significantly less anxiety, greater academic performance, and greater career choice satisfaction [14]. However, this study used professionally qualified post-graduate mentors rather than peer or near-peer mentors, resembling more closely the relationship between student and teaching assistant, which is more instructional in nature. Another study, using first-year psychology students in the USA, suggested that PAL interventions improve exam performance [15]. However, this study was quasi-experimental, without randomised allocation of participating students to control groups, and only examined effects of peer mentoring in a single discipline. To assess whether particular NPAL/PAL interventions have benefits across different disciplines and courses, a single format of delivery should be assessed across different programmes. Finally, Micari and Pazos conducted a longitudinal study involving undergraduate STEM and social science students participating in weekly near-peer-led study groups at a different US university, using a robust case–control matching approach to compare academic outcomes between participant and non-participant groups over three academic years [16]. Grade performance was significantly higher for the NPAL groups compared to the non-NPAL control, suggesting that NPAL engagement had a positive effect on academic performance. However, the opt-in allocation of students to the group-study programme and the absence of a within-participant comparison approach meant that the reported benefit to academic attainment may have been influenced by other unmeasured characteristics of the participating students. Overall, there remains a need for robust, experimental, quantitative evidence regarding the effectiveness of peer support strategies and learning programmes in promoting academic attainment and other positive outcomes for participating students across different disciplines in higher education.

Here we present new experimental evidence from an RCT study of NPAL programmes in two different disciplines at our University. The aim of our study was to assess whether participation in NPAL causally affects academic attainment and student confidence within higher education. To capture academic attainment, we assessed impacts on both response accuracy and response time, two common measures of cognitive processing that influence performance in time-limited tests but are traded off against each other [17, 18]. We hope to offer novel insights that might help direct and underpin developments in (N)PAL across the global higher education sector.

## Material and Methods

### Participants

All near-peer mentors (*n* = 15) and study participants (*n* = 32) were undergraduate students at the University of Exeter during the 2018/2019 academic year. The participants were first-year students enrolled on either the BSc Medical Sciences degree (in the College of Medicine and Health) or on the BSc Psychology degree (in the College of Life and Environmental Sciences). All participants were recruited via volunteer sampling, in which the NPAL programme organiser attended the introductory lecture for the module and advertised the study. Across the two-degree programmes, 86 participants volunteered to take part, but 54 of those either did not attend any of their NPAL sessions or did not attempt the two weekly quizzes. Of the 32 participants who provided usable data, 15 were from BSc Medical Sciences, 17 from BSc Psychology; seven (22%) were male, 25 (78%) were female; and their ages ranged from 18 to 42 years (M_age_ = 20, SD_age_ = 3.5). The study participant demographics were similar to those of the wider cohorts from which they were recruited. All participants who completed the study received a monetary £10 gift voucher in recognition of their time and participation upon completing a minimum of three assigned workshops and six multiple-choice tests.

All near-peer mentors recruited to NPAL programmes attended workshops that provided training in basic mentoring skills, including lesson planning, evaluation, good practice in mentee interaction, safeguarding and setting professional boundaries. The students in both degree programmes were in Stage 2 (Level 5: UK Qualification Framework as set by the Quality Assurance Agency for Higher Education) or Stage 3 (Level 6), having previously completed the taught modules in this study during their Stage 1 of their respective degrees. All mentors completed additional study-specific training to equip them with the skills to deliver the NPAL sessions in a consistent manner for the trial. The study-specific sessions outlined the study protocol and provided instructions on how to facilitate the sessions using techniques such as Socratic questioning and prompting, rather than providing straight answers or engaging in direct teaching [19]. All mentors received monetary remuneration for training and delivery of study sessions.

### Design

We administered the NPAL sessions across both disciplines using an RCT design. Participants were provided with an anonymised participant ID upon volunteering for the study and were randomly allocated to complete each of four protocol conditions within their taught course across a 4-week period from January to March 2019. In each week, participants attended their normally timetabled academic-led lectures, plus one of the following treatment conditions: (1) an introductory NPAL session prior to the academic-led lecture; (2) a review NPAL session after the academic-led lecture; (3) both introductory and review NPAL sessions; (4) no NPAL sessions. We used a within-participants design such that participants experienced all these conditions across the four weeks, with the order of the conditions randomised and counterbalanced across the sample to ensure there were equal numbers of participants in all groups.

### Quiz Performance

Prior to the introductory NPAL session each week (and regardless of subsequent attendance at that session), all study participants were asked to complete an online quiz (hereafter, Quiz 1). Quiz 1 comprised 10 multiple-choice questions (MCQs; choose one correct answer from four or five options) on the topic covered in that week’s academic-led lecture, with participants allowed up to 1 min to answer each question. A 1-min response restriction was applied to reflect the timed exam conditions of the final assessment for the module, and to restrict the likelihood that students used lecture materials to answer the questions, maintaining ‘closed book’ assessment. The quiz was implemented via Qualtrics, with responses selected using mouse clicks. The Qualtrics platform automatically recorded the timing of the participant’s first mouse click for each question, reflecting the speed with which they first chose an answer. For each question, participants were also asked to self-report their confidence that the answer they provided was correct on a 5-point scale from 1 (“guess”) to 5 (“certain”). The same quiz was repeated for all participants after the academic-led lecture and review NPAL session had taken place (hereafter, Quiz 2), allowing us to measure changes in test scores, response speed and self-reported confidence in answering the topic questions. Quiz questions were constructed by the module lead in each programme using the lecture materials from that week. The MCQ format follows the same format as the final exam on the module, and questions in the weekly quiz reflected the approximate difficulty of final exam items.

### NPAL Sessions

The introductory NPAL sessions occurred prior to the academic-led lecture session and consisted of a 60-min session, designed to promote open discussions around the lecture content material for each week. General context around the lecture topic was provided, including use of TED Talks to give an overview of the topic to participants. TED talks were selected as learning material as they provide an example of how to engage in learning outside of lectures, as they are freely available learning resources [20]. Students have been shown to enjoy engaging with TED talks as part of their curriculum, stating they may provide a more personal lens to topics, and encourage engagement in independent research outside of formal lecture sessions [21]. Each introductory NPAL session TED talk was selected by the NPAL programme lead in each course to align with the overall focus of the coming week’s lecture. In the psychology programme, all introductory NPAL sessions were constructed by the NPAL lead (BM-P), with available mentors volunteering to facilitate student discussions during the session. Following general context, students were asked a series of informal questions and were encouraged to engage in group discussions amongst themselves, and with the mentors. The review NPAL sessions occurred following the academic-led lecture each week and consisted of a 60-min session designed to recapitulate the lecture material from that week. In the psychology programme, the NPAL lead posted a poll to mentors each week segmenting the weekly lecture slides into subtopics for them to prepare material on. The NPAL lead always delivered at least one subtopic and was present at every session. Mentors submitted their prepared material to the NPAL lead at least three days ahead of the review session to allow for checking and editing if required. The review sessions used both exam-style MCQs and short-answer questions (SAQs) which were discussed in groups to consolidate learning. The mentors organised the teaching material as well as the structure of the taught sessions for both the introductory and the review NPAL sessions. These session materials and lesson structure were reviewed by a senior academic (the module lead) prior to delivery to ensure consistency and accuracy of the taught content. In psychology, all mentors received and conducted an informal peer-to-peer assessment of their mentoring and were provided verbal feedback from the NPAL lead based on this assessment and their general performance. A ‘debrief’ was conducted after each NPAL session to discuss what went well, and what could be done differently in future weeks. However, it should be noted no formal fidelity or quality assessments were conducted.

### Weekly Evaluation of NPAL Sessions

At the end of each study week, following Quiz 2, participants were sent a weekly evaluation questionnaire. The questionnaire contained a series of single-item questions regarding each NPAL session (e.g., “The Introductory (pre-session) NPAL session made me feel more confident going into that week's lectures”) and the experience of NPAL sessions overall (e.g., “The resources used in the NPAL sessions [i.e., PowerPoint slides] were effective”) rated on 5-point Likert scales from 1 (“strongly disagree”) to 5 (“strongly agree”). Two open-text response questions were included to collect qualitative data on why participants felt the NPAL sessions being student-led was important and provide general feedback for the mentors. The qualitative data generated from the open-text responses were not analysed further as most participants did not provide feedback, thus there were insufficient data for saturation to allow for analysis.

### Ethics

Full ethical approval for this work was granted by the University of Exeter Psychology Ethics Committee (approval reference eCLESPsy000179). All participants were given a project information pack and completed an informed consent form prior to beginning the study. In accordance with ethics guidelines, each participant was assigned a participant study ID number to preserve their anonymity. All participants were reminded that their participation was voluntary and that they could opt out at any time without their programme grade being affected.

### Data Analysis

To analyse the data, we used linear mixed-effects models (LMMs) and generalised linear mixed-effects models (GLMMs). Mixed-effects models (also known as multilevel models) are increasingly used in many fields, including medical sciences [22] and psychology [23], to model data that contain clusters of non-independent observations, as is the case in repeated-measures designs. For our study, where the multiple observations (e.g. weekly quiz scores) of each participant cannot be considered independent, (G)LMMs provide a powerful framework that makes full use of the data by modelling all observations and their correlations within participants, rather than losing information by collapsing them to aggregate or average measures. Compared to alternative approaches such as repeated-measures analysis of variance, mixed-effects models do not exclude participants with partially missing data (common in RCTs) and can accommodate categorical and continuous predictors at multiple levels of the hierarchical design. Furthermore, as an extension to LMMs, GLMMs incorporate link functions to model discrete data that do not follow a Gaussian (i.e. normal) error distribution, such as binary outcomes (e.g. whether a quiz question was answered correctly). For more detailed information, see Snijders & Bosker book on multilevel analysis [24].

Prior to analyses, data from any participants who failed to attend the NPAL session(s) they had been assigned to, or who attended one or more sessions they had not been assigned to, were removed to ensure that this self-selecting sample could not bias the results. We then conducted three main analyses. First, to investigate the effect of NPAL session attendance on quiz performance, we fitted a GLMM with a binomial error structure, implemented using the *lme4* package version 1.1–35-5 in R version 4.4.1 (R Core Team, 2017) [25]. The outcome of interest was a binary variable indicating whether the response to each question in Quiz 2 was correct (1) or incorrect (0). Modelling each question separately is preferable to using an aggregate or average quiz score, because it uses more of the available data, efficiently accounts for participant variability in the number of questions answered, and allows us to model the extent to which answers to the same question are correlated across participants. We modelled this binary correct/incorrect outcome as a function of the following fixed effects: introductory NPAL session attendance (1 = attended, 0 = did not attend), review NPAL session attendance (1 = attended, 0 = did not attend), week (1–4, treated as a categorical factor, with Week 1 as the reference category), degree programme (Medical Sciences or Psychology, with Medical Sciences as the reference category following R’s default alphanumeric ordering) and whether the participant’s response to the same question in Quiz 1 was correct (1) or incorrect (0). We had no prior hypotheses regarding the effects of week and degree programme but included these terms to account for varying quiz difficulty across weeks and differences between Medical Sciences and Psychology students. We also included a two-way interaction term between week and degree programme, to capture differences between Medical Sciences and Psychology in the changing difficulty of content across the four weeks, and a two-way interaction term between the effects of attending the introductory and review PAL sessions, in case these were synergistic (e.g. the review session might be more effective when a participant also attended the introductory session in that same week). These interaction terms were removed if they did not significantly improve the fit of the model. Participant identity and quiz question were included as randomly varying intercepts to account for non-independence in the data. We evaluated the significance of all fixed effects using likelihood-ratio (LR) tests, which compare the deviance (− 2 × log-likelihood) between models with and without that predictor. Second, to assess whether NPAL session attendance affected response speed, we analysed variation in the time (in seconds) to the first click, using LMMs with a Gaussian error structure and the same set of fixed effects as before (introductory NPAL session attendance, review NPAL session attendance, week, degree programme, whether the participant responded correctly to the same question in Quiz 1, plus interaction terms week × degree programme and introductory × review NPAL session attendance), with randomly varying intercepts for participant identity and quiz question. The distribution of click times was strongly right-skewed, so was square-root transformed prior to analysis to ensure normality and homoscedasticity of the residuals. Finally, to examine variation in self-reported confidence (rated 1–5) for each question, we fitted an LMM with a Gaussian error structure and the same set of fixed effects, again with randomly varying intercepts for participant identity and quiz question.

## Results

### Participation Across Weeks and Treatment Conditions

Of the 86 participants who originally registered, 53 (62%) correctly attended their assigned NPAL sessions in Week 1. Thereafter we saw a modest drop in participation, with 46 (53%) attending correctly in Week 2, 45 (52%) in Week 3 and 42 (49%) in Week 4. Engagement with the weekly quizzes was lower but did not show a steady decline: 27 participants (31%) attempted both Quiz 1 and Quiz 2 in Week 1, 24 (28%) in Week 2, 28 (33%) in Week 3 and 26 (30%) in Week 4.

Participation across the four treatment conditions is summarised in Table 1.

**Table 1:** Summary of participation across the four treatment conditions

	Total length of participation (number of weeks)												Total number of participants providing data for that condition
	4 wks	3 wks			2 wks					1 wk			
Treatment condition													
None (N)	N	N	N	N	N	N	N			N			26
Introductory (I)	I	I	I		I			I			I		24
Review (R)	R	R		R		R			R			R	19
Both (B)	B		B	B			B	B	B				24
Number of participants													
(a) attending correctly	26	3	2	1	6	2	2	0	0	44	0	0	
(b) attending correctly and completing both quizzes	15	0	5	0	1	1	2	1	1	2	2	2	32

Data were provided by those participants who (a) followed the correct attendance pattern in that week and (b) completed both Quiz 1 and Quiz 2. The letters N, I, R and B are shown if data were provided for that treatment condition and left blank otherwise

Of the 86 participants who originally registered, 42 (49%) attended at least one of their assigned NPAL sessions, while the remaining 44 (51%) failed to attend any of them. Of those 42 attenders, 26 (30% of the original sample) attended their assigned sessions correctly in all four weeks of the study, a further six (7%) attended correctly in three of the four weeks, and the remaining 10 (12%) in two of the four weeks. However, only 32 of the attending participants (37% of the original sample) completed both quizzes (Quiz 1 and Quiz 2) in one or more weeks, allowing us to measure the impact of their allocated NPAL session(s): 15 (17%) attended correctly and completed both quizzes in all four weeks, five (6%) did so in three of the four weeks, six (7%) did so in in two of the four weeks and the remaining six (7%) did so in just one week. Overall, this gave us data for 26 participants in the week they attended no NPAL sessions, 24 in the week they attended the introductory session only, 19 in the week they attended the review session only and 24 in the week they attended both sessions.

### Quiz Performance: Correct Answers

In the GLMM, the interaction term between attendance at the introductory and review NPAL sessions did not significantly predict the chance of answering correctly (LR test: *χ*^2^(1) = 0.05, *p* = 0.830), so this term was removed from the model. The estimated effects and significance tests for the other predictors are shown in Table 2.

**Table 2:** Estimates of fixed effects in a binomial GLMM predicting quiz performance (log odds of answering a question correctly) after attending (vs. not attending) introductory and review NPAL sessions, with random effects of participant ID (*n* = 32) and quiz question (*n* = 80)

Fixed Effect	Estimate ± s.e	*χ*^2^ †	d.f	*p*
Intercept*	− 0.318 ± 0.350	–	–	–
Introductory NPAL session	**0.361 ± 0.162**	**4.9**	**1**	**0.027**
Review NPAL session	0.154 ± 0.174	0.8	1	0.381
Quiz 1 performance	**1.216 ± 0.169**	**51.7**	**1**	** <. 001**
Week × degree programme	**–**	**18.2**	**3**	** < 0.001**

The overall model fit was highly significant, compared to a null model with no fixed effects (ΔAIC = 70.4; *χ*^2^(10) = 90.4, *p* < 0.001)

*Predicted log odds of answering correctly in Week 1 for a Medical Sciences student who did not attend either NPAL session and got the associated question wrong in their previous attempt (Quiz 1)

†Change in deviance from a likelihood-ratio test comparing models that include or omit that predictor

**Fig. 1:**
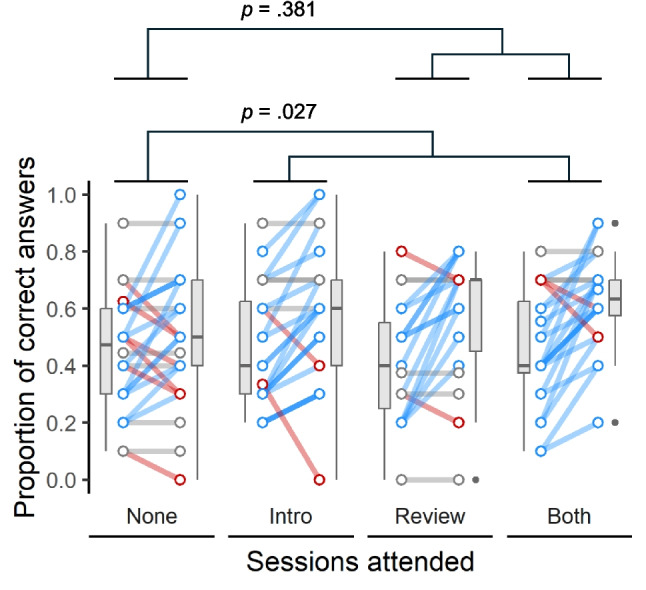
Proportion of correct answers on the same set of 10 multiple-choice questions, both before (Quiz 1; left point of each pair) and after (Quiz 2; right point) attending neither of the NPAL sessions (None), the introductory NPAL session only (Intro), the review NPAL session only (Review) or both NPAL sessions (Both). Coloured lines connect the before and after points for each individual participant, showing either an improvement (blue), decline (red) or no change (grey) in performance between the two attempts. Boxplots depict the median, interquartile range (IQR) and outliers, with whiskers extending to 1.5 times the IQR beyond the edge of the box. Compared to participants who attended neither session (None), the improvement was significantly greater for those who attended the introductory session (Intro and Both groups; *p* = 0.027), but not for those who attended the review session (Review and Both groups; *p* = 0.381)

As expected, there was a strong association between performance in the first and second attempts at the same quiz, whereby participants were significantly more likely to answer correctly in Quiz 2 if they had answered the same question correctly in Quiz 1 (*p* < 0.001). There was also a significant difference overall between Medical Sciences and Psychology students in how their performance changed across the four weeks of the study (*p* < 0.001). Controlling for these effects, participants who attended the introductory NPAL session performed significantly better than those who did not attend (*p* = 0.027; Fig. 1), increasing their odds of answering correctly by over 40% (odds ratio 1.43, 95% CI 1.04–1.97), whereas there was no significant improvement for those who attended the review NPAL session (*p* = 0.381; Fig. 1).

### Response Speed

Average response speeds were quicker for questions that more participants answered correctly, with a shorter time to the first click (Pearson correlation: *r* = − 0.383, *t* (78) = − 3.66, *p* < 0.001). In the LMM predicting time to the first click, the interaction term between attendance at the introductory and review NPAL sessions was not significant (LR test: *χ*^2^(1) = 0.81, *p* = 0.367), so this term was removed. The estimated effects and significance tests for the other predictors are shown in Table 3.

**Table 3:** Estimates of fixed effects in an LMM predicting response time (number of seconds to the first mouse click, square root transformed) after attending (vs. not attending) introductory and review NPAL sessions, with random effects of participant ID (*n* = 32) and quiz question (*n* = 80)

Fixed Effect	Estimate ± s.e	*χ*^2^ †	d.f	*p*
Intercept*	3.327 ± 0.230	–	–	–
Introductory NPAL session	** − 0.144 ± 0.073**	**3.8**	**1**	**0.050**
Review NPAL session	0.044 ± 0.079	0.3	1	0.576
Quiz 1 performance	− 0.077 ± 0.077	1.1	1	0.304
Week × degree programme	**–**	**14.7**	**3**	**0.002**

The overall model fit was significant, compared to a null model with no fixed effects (ΔAIC = 16.7; *χ*^2^(10) = 22.2, *p* = 0.014)

*Predicted response time (seconds, square root transformed) in Week 1 for a Medical Sciences student who did not attend either NPAL session and got the associated question wrong in their previous attempt (Quiz 1)

†Change in deviance from a likelihood-ratio test comparing models that include or omit that predictor

**Fig. 2:**
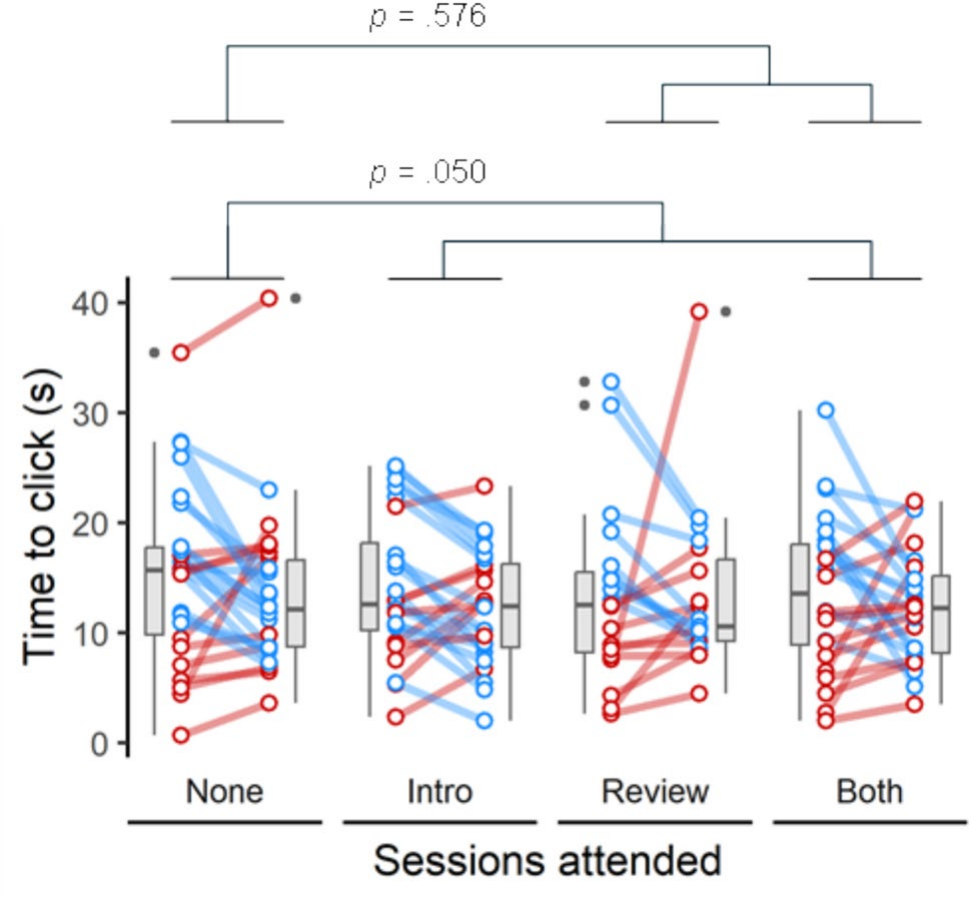
Response time (number of seconds to first mouse click) averaged across the same set of 10 multiple-choice questions, both before (Quiz 1; left point of each pair) and after (Quiz 2; right point) attending neither of the NPAL sessions (None), the introductory NPAL session only (Intro), the review NPAL session only (Review) or both NPAL sessions (Both). Coloured lines connect the before and after points for each individual participant, showing either a decrease (blue), increase (red) or no change (grey) in response time between the two attempts. Boxplots depict the median, interquartile range (IQR) and outliers, with whiskers extending to 1.5 times the IQR beyond the edge of the box. Compared to participants who attended neither session (None), the reduction in response time was significantly greater for those who attended the introductory session (Intro and Both groups; *p* = 0.050), but not for those who attended the review session (Review and Both groups; *p* = 0.576)

Participants who had answered correctly in their previous attempt (Quiz 1) did not click more quickly than those who had not (*p* = 0.304). The change in response speed across the four weeks of the study differed significantly between Medical Sciences and Psychology students (*p* = 0.002). Controlling for these effects, participants who attended the introductory NPAL session were significantly quicker to respond than those who did not attend (*p* = 0.050; Fig. 2), making their first click 0.9 s earlier (95% CI 1.8–0.0), whereas attendance at the review NPAL session did not affect this response time (*p* = 0.576; Fig. 2).

### Student Confidence

Across questions, the average self-reported confidence score was positively correlated with the proportion of participants answering that question correctly (*r* = 0.715, *t* (78) = 9.02, *p* < 0.001). In the LMM used to explain variation in confidence scores, there was again no significant interaction between attendance at the introductory and review NPAL sessions (LR test: *χ*^2^(1) = 1.53, *p* = 0.216), so this term was removed. The estimated effects and significance tests for the other predictors are shown in Table 4.

**Table 4:** Estimates of fixed effects in an LMM predicting self-reported confidence (rated 1–5) after attending (vs. not attending) introductory and review NPAL sessions, with random effects of participant ID (*n* = 31) and quiz question (*n* = 80)

Fixed Effect	Estimate ± s.e	*χ*^2^ †	d.f	*p*
Intercept*	3.241 ± 0.255	–	–	–
Introductory NPAL session	**0.214 ± 0.078**	**7.5**	**1**	**0.006**
Review NPAL session	0.091 ± 0.087	1.1	1	0.289
Quiz 1 performance	**0.260 ± 0.083**	**10.0**	**1**	**0.002**
Week × degree programme	**–**	**28.1**	**3**	** < 0.001**

The overall model fit was highly significant, compared to a null model with no fixed effects (ΔAIC = 20.6; *χ*^2^(10) = 57.5, *p* < 0.001)

*Predicted self-reported confidence in Week 1 for a Medical Sciences student who did not attend either NPAL session and got the associated question wrong in their previous attempt (Quiz 1)

†Change in deviance from a likelihood-ratio test comparing models that include or omit that predictor

**Fig. 3:**
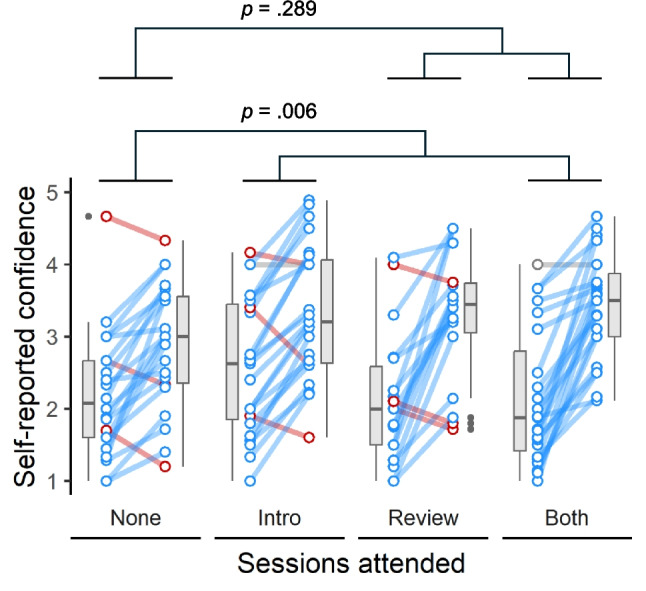
Self-reported confidence (on a scale from 1 to 5) averaged across the same set of 10 multiple-choice questions, both before (Quiz 1; left point of each pair) and after (Quiz 2; right point) attending neither of the NPAL sessions (None), the introductory NPAL session only (Intro), the review NPAL session only (Review) or both NPAL sessions (Both). Coloured lines connect the before and after points for each individual participant, showing either an increase (blue), decrease (red) or no change (grey) in confidence between the two attempts. Boxplots depict the median, interquartile range (IQR) and outliers, with whiskers extending to 1.5 times the IQR beyond the edge of the box. Compared to participants who attended neither session (None), the increase in confidence was significantly greater for those who attended the introductory session (Intro and Both groups; *p* = 0.006), but not for those who attended the review session (Review and Both groups; *p* = 0.289)

Self-reported confidence scores were higher in participants who had answered correctly in their previous attempt (Quiz 1; *p* = 0.002). The pattern of changes in self-reported confidence across the four weeks of the study differed significantly between Medical Sciences and Psychology students (*p* < 0.001). Controlling for these effects, participants who attended the introductory NPAL session reported significantly higher confidence than those who did not (*p* = 0.006; Fig. 3), whereas there was no detectable confidence boost for those who attended the review NPAL session (*p* = 0.289; Fig. 3).

### Weekly Evaluation of PAL Programmes

A total of 63 separate participant responses to the weekly evaluation questionnaires were recorded across both studies (summarised in Table 5).

**Table 5:** Summary of qualitative data obtained from weekly evaluation questionnaires, comprising a series of Likert questions (Questions 1–5, *n* = 63) plus one question (Question 6, *n* = 40) to identify which NPAL session participants found most useful

Question	Agree/Strongly agree	Neither agree nor disagree	Disagree/Strongly disagree
	No. (%)	No. (%)	No. (%)
1. The introductory PAL session made me feel more confident going into that week's academic-led classes	36 (57%)	24 (38%)	3 (5%)
2. The introductory PAL session helped me to prepare for that week’s academic-led classes	40 (63%)	20 (32%)	3 (5%)
3. The review PAL session helped me to understand the content covered in that week’s academic-led classes	46 (73%)	15 (24%)	2 (3%)
4. Knowing that the PAL sessions were entirely student-led was important to me	21 (33%)	22 (35%)	20 (32%)
5. The peer mentors were effective at engaging me in the content	47 (75%)	13 (21%)	3 (5%)
	Introductory	Review	Both
6. Most useful PAL session*	8 (20%)	17 (43%)	15 (38%)

*The response options were as follows: “I found the introductory PAL session the most useful” (Introductory), “I found the review PAL session the most useful” (Review) or “Having both an introductory and a review PAL session was effective (i.e., better than having either alone)” (Both)

Participants reported that attending the introductory NPAL session improved their confidence and helped them to prepare for upcoming academic-led classes (57% and 63% agreeing or strongly agreeing, respectively). 73% of respondents agreed or strongly agreed that the review NPAL sessions helped their understanding of content covered in academic-led classes. Overall, 41% of respondents found the review NPAL sessions to be the most useful, with 20% identifying the introductory NPAL sessions as such and 37% stating that having both an introductory and a review NPAL session each week was preferable to having either alone. 75% of respondents agreed or strongly agreed that the mentors were effective at engaging them in the NPAL sessions.

In summary, attending an introductory NPAL session had significant positive effects on quiz performance, response speed and self-reported confidence, while attending a review NPAL session did not affect any of these measures. By contrast, participant perceptions of the usefulness of attending these sessions, as captured in the weekly evaluation questionnaires, indicated that, overall, participants felt the review NPAL sessions were the most useful, suggesting a mismatch between the perceived usefulness and the actual effectiveness of the NPAL sessions.

## Discussion

To investigate the effectiveness of NPAL programmes, we designed and implemented a randomised control trial involving first-year undergraduate student participants attending student-led NPAL sessions for two different degree programmes (in two different colleges) at our University. Our results showed that participation in these academically focussed NPAL programmes resulted in measurable and statistically significant improvements in weekly test scores, response speed and self-reported confidence in academic abilities. Whilst attendance at the sessions decreased, overall confidence and engagement of the remaining students increased over the four-week study period. This allowed the students who engaged most in the sessions to get enhanced small group learning exposure with their student mentors. Small-group learning may provide an opportunity for students to engage collaboratively in learning, with facilitators encouraging students to interact with each other to achieve a shared learning goal [26]. Future studies may seek to assess whether different group sizes affect the efficacy of (N)PAL sessions.

The inclusion of both introductory and review NPAL sessions represented a novel approach to the delivery of (N)PAL programmes within our institution and, as far as we are aware, is an approach to (N)PAL that has not previously been reported in the literature. The introductory (N)PAL sessions differ from traditional ‘previewing’ approaches [27] as they did not rely on students independently reviewing information prior to sessions. The sessions were delivered in a collaborative, group format, using resources such as TED talks to facilitate discussion and ‘pre-learning’. We were particularly interested to see how the introductory NPAL sessions performed in terms of preparing participants for subsequent academic-led classes. Within our institution and similar higher education institutions that provide academic support through peer support schemes, (N)PAL programmes tend to be offered as a supplementary intervention either following the delivery of an academic-led class or as a pre-assessment revision tutorial [28]. Our model differed by offering both formats of support, and incorporated pre-learning ahead of lecture content, rather than at the pre-assessment stage prior to final exams. In our model of academic support, the introductory NPAL sessions were designed to allow students to share prior knowledge of that week’s forthcoming academic-led classes and to promote a shared understanding of the intended learning outcomes prior to attending class.

## The Introductory NPAL Session

In our study, we found that, based on weekly quiz performance, participants across both degree programmes who attended the introductory NPAL session saw a significant improvement in their weekly test score compared to those who did not, and this same outcome was also observed in their response speed and self-reported confidence scores. The qualitative data collected from weekly evaluation survey questionnaires (*n* = 63) also suggested a positive effect of the introductory NPAL session, with 57% of respondents agreeing or strongly agreeing that the introductory session made them feel more confident going into subsequent academic-led classes, and 63% similarly stating that the introductory session helped them to prepare for subsequent classes.

## The Review NPAL Session

As with the provision of preparatory content described earlier, the process of consolidating learning through attendance at post-class tutorials is another approach widely used throughout higher education and replicated here in our study’s review NPAL sessions. However, although attendance at the review NPAL session was perceived by participants as being more useful than the introductory sessions, we found no evidence that the former led to an improvement in weekly test scores, response speed or confidence scores. We also found no significant interaction between the effects of attending the introductory and review NPAL sessions, indicating that there was no extra benefit from attending both sessions during the same week beyond the sum of their separate effects.

The review NPAL session provided an opportunity for students who had attended that week’s academic-led classes to consolidate their learning, through sharing knowledge and reviewing whether they had met the intended learning outcomes. In this way, the review NPAL sessions in our study closely resemble the traditional tutorial system, whereby students undertake set activities as part of a small group guided by a tutor that link back to the content that they have already been exposed to during larger-format classes, such as lectures [29]. The prevalence of this type of educational model may go some way to explaining why the students perceived the review sessions as more useful than the less conventional pre-lecture/workshop introductory sessions, even though the latter were better attended and, unlike the review sessions, yielded significant improvements in weekly test scores, response speed and self-reported confidence. Familiarity with learning and educational approaches may enhance meta-cognitive awareness (MA) in students, given MA relies on previous experience [30]. Through an enhanced understanding of their own cognitive processes, understanding how a particular session format may contribute to their own planning and regulation of learning, may in turn enhance predicted benefits of this approach [31]. As students have previously engaged in the review NPAL format, this may have contributed to their perception of these sessions as more useful, as they use past knowledge of review-format sessions enhancing learning to predict future outcomes. This may offer insight into why the ‘novel’ introductory NPAL sessions were rated as less useful, as students did not have prior knowledge to interpret the perceived benefits of this session.

## Implications

There are several lines of evidence that could offer an explanation as to why participants who attended the introductory NPAL sessions in our study performed better in all measured indices. The setting of preparatory work to complete before attending class is a well-established pedagogical approach and has gained wider popularity within the higher education sector over the past two decades, with growing utilisation of the ‘flipped’ or ‘inverted’ classroom model across many disciplines [32]. Although the intention of this approach is to promote active learning and foster a more discursive and questioning learning environment within the classroom, the reality is often less satisfactory. In our experience and in that reported by others [33], the main limitation with this approach is that, for it to be effective, students need to arrive at class having already read or completed the preparatory work.

In contrast to the typical inverted classroom approach, where students are required to engage with preparatory content independently, our model of peer support involves students meeting as a group within the introductory NPAL sessions and participating in the collaborative sharing of prior knowledge, expectations, and the intended learning outcomes of the upcoming class. Such a collaborative, peer-led, small-group learning environment has previously been shown to be an effective approach to improve grade performance and student retentions, particularly amongst students with lower prior attainment scores [16]. Thus, providing the opportunity for students to engage in more social preparatory activities prior to attending class is one possible way to improve engagement with the inverted classroom approach and realise its evident potential in enhancing educational attainment. This is pertinent to undergraduate programmes, such as the two studied in this paper. In our view, such a preparatory approach is ideally suited to student-led (N)PAL programmes, which carries the added benefit of alleviating the time burden that would otherwise be placed on academic teaching staff.

Our study investigated the short-term effects of an NPAL programme on academic performance, evaluating the academic outcome of students through our own formative tests. We did not assess final exam scores through the summative assessment’s exams. Comparing final exam scores of participants in the different NPAL conditions would be a valuable avenue for future work, to determine how long the benefits we measured persist beyond the 4-week period and evaluate whether they translate into higher academic achievement. Additionally, comparing outcomes from sessions led by students versus junior tutors (trained educators) versus more senior tutors could elicit deeper insight into the benefits that peer, and near-peer teaching programmes have on academic achievement. This could be conducted through a staged crossover design, to ensure all participants experience and can compare all three types of tutors. This study method could be compared to students studying alone or committing the same amount of time on an online learning software, to help identify the impact of independent versus collaborative learning.

A potential limitation of the present study is that, because participation was voluntary, the majority of participants did not attend all of their timetabled NPAL sessions or complete all of the quizzes (Table 1). Participants who attended more sessions and completed more quizzes, and who therefore contributed more data, may be inherently more motivated students; thus, our findings may predominantly represent those students who are most likely to be benefit from NPAL programmes, due to their willingness to engage with additional study support. While the RCT design, with random allocation and counterbalancing of condition allocation, ensured that the differences we observed between conditions were valid, it is plausible that the positive effects of NPAL attendance may be most apparent for students motivated to attend across the semester. Thus, strategies to promote consistent student engagement in (N)PAL programmes should be considered in future research. Additionally, despite using the same procedure to construct weekly quizzes as the final exam (i.e., module lead constructs questions based on content delivered in lectures), no formal reliability or validity analyses of the quizzes administered in the present study were conducted. Future studies using weekly assessments may aim to assess the properties of weekly quizzes to provide comment on the rigor of this method to assess student performance.

Finally, due to logistical constraints, this study was restricted to two-degree programmes within one university, and such we acknowledge the limited scope of our conclusions. To extrapolate the results more widely, we encourage colleagues at other higher education institutions to replicate our RCT design across a wider range of degree programmes.

Based on the findings from our study, it would be interesting to explore further whether, across different disciplines and institutions, there is a measurable difference in educational outcomes between student cohorts who are encouraged to engage actively with preparatory resources versus those who instead rely solely on attending post-class consolidative activities, such as tutorials. Addressing the apparent mismatch between students’ perception of the value of attending a class (as measured using qualitative self-report surveys) and the measurable benefit they receive from doing so (as measured using quantitative data on academic test performance) is something that requires careful consideration if student engagement with future (N)PAL programmes within our own institution (and others) is to be sustained.

## Conclusion

Our data provide robust experimental evidence that the engagement of undergraduate students with NPAL programmes causally leads to measurable improvements in their weekly learning, response speed and confidence. This supports the suggestion that (N)PAL programmes are an important adjunct to the conventional academic-led teaching and learning environments offered by higher education institutions. In the current climate of funding deficit and financial restraint within the UK’s higher education sector, it has never been more important to support education funding applications with solid, quantifiable evidence of impact. The data presented in this paper build on previous quantitative studies and go some way towards achieving this aim, providing strong evidence for the multi-faceted benefits of (N)PAL programmes, as well as a framework for other educational institutions to evaluate their own (N)PAL programmes. A valuable direction for future research would be to conduct a larger-scale, cross-sector/multi-institutional study, to provide an even stronger evidence base for the effect that (N)PAL programmes have on student academic attainment.

## Data Availability

All data generated or analysed during this study are included in this published article and its supplementary information files.

## Abbreviations

*GLMM*: Generalized Linear Mixed-effects Model
*LMM*: Linear Mixed-effects Model
*MCQ*: Multiple Choice Question
*PAL*: Peer-Assisted Learning
*NPAL*: Near-Peer-Assisted Learning
*RCT*: Randomised Control Trial
